# The National Poor-Law Officers' Association

**Published:** 1917-03-17

**Authors:** 


### The National Poor-Law Officers' Association.

WE have received a long letter from Mr. C. A. W. Roberts, Master of Walton Institution, that is an infirmary with 130 nurses under the control of the workhouse of which Mr. Roberts is the master. This letter deals with points already covered, and the pressure on our space makes it impossible to find room for the whole letter this week.

Mr. Roberts makes (1) a claim that the N.P.L.O.A.'s action caused the College to adopt the L.G.B. standard, whereas this standard was in fact accepted by the College when the L.G.B.'s amended standard was formulated; (2) that there is a danger of the individual training certificates of each training-school being accepted for all time. In fact there is not, and never has been, any such danger, standardisation being provided for when the College is organised. Class distinction in this connection is a myth created by the N.P.L.O.A.; (3) there is no basis which will bear examination on which to rest the misleading argument that Poor-Law representation on the College Council will be one in 39 members. The College Bill provides that two-thirds of the whole Council shall be elected by the nurses on the General Register.

Mr. Roberts' letter concludes :—

"If I or those working with me felt for one moment that our interest in this movement would accentuate the difference between Poor-Law and general-trained nurses, we would say not another word, but reason tells us that the bias against everything Poor-Law, as shown by the College authorities and your paper, can only mean that when the great changes come, as come they will after the war, unless the Poor-Law nurses have a strong organisation to watch their interests, instead of a fusion of public health authorities, we shall see an absorption, and this great Nursing Service which has proved itself over and over again to be one of the greatest assets to the country still 'waiting to see.'"

We are bound to accept this statement as definite and sincere. We hope, therefore, that the National Poor-Law Officers' Association will at once discontinue its agitation and pressure on the Poor-Law nurses, seeing that it must, if continued, "accentuate the difference between the Poor-Law and general-trained nurses." Mr. Roberts' reasoning is at fault, for the College authorities, we are confident, have no such bias as he claims. Further, THE HOSPITAL has no bias either, as its columns demonstrate, wherein for years it has consistently advocated the reconstitution of the whole of the Poor-Law Infirmaries as national hospitals, a much-desired reconstruction of hospital accommodation after the war, in this country, which we have every reason to believe may become speedily an accomplished fact.—ED. THE HOSPITAL.

